# Supplementary material for: Sex differences in patch-leaving foraging decisions in rats
Source: Oxf Open Neurosci. 2023 Oct 17;2:kvad011. doi: 10.1093/oons/kvad011 (PMC11003400; doi:10.1093/oons/kvad011)
Supplement: Web_Material_kvad011 [file Web_Material_kvad011.zip › r2r.docx]

We thank the reviewers for their attention to our manuscript and for their helpful suggestions for improvement. Below are point-by-point responses to reviewer’s inquiries and a description of the changes we have made to the manuscript.

Reviewer: 1

Comments to the Author

In this study the authors have developed a novel foraging task designed to more closely emulate foraging patterns that occur in the wild. The new task is interesting and has the potential to assess the role of movement and spatial navigation in the neural underpinnings of foraging behavior. However, I have an issue with the statistics that I believe the authors need to address before this can move to publication. This and other issues are listed below.

1. I am not sure if the authors used the appropriate statistics in this paper. The authors report that 10 rats total were used in the study, 5 male and 5 female. However, my worry is that they used each rat's data point per session as an independent "subject" in their analysis. This fear is mostly rooted in the apparent degrees of freedom they report. For example, on page 11 of the manuscript, the authors report a t-test with 357 degrees of freedom, when there should only be 8 degrees of freedom (assuming 5 rats of each sex). Fig. 5C, with which this finding is associated, seems to have enough dots to lead to 357 degrees of freedom, but each dot is a session that the rat ran, not an individual rat's data. Similar usage of statistics appears elsewhere in the paper. If the authors want to analyze each time point, then they should run a repeated measures ANOVA that includes each manipulation for each of the 10 rats. It is not appropriate to use each data point as an independent subject that is then analyzed in a t-test; doing so artificially reduces the p-value. The authors should either redo their statistics appropriately or justify why they have so many degrees of freedom.

We thank the reviewer for pointing this out. We agree that the comparisons that relied on t-tests did not account for repeated measurements in the same subject. To remedy this, we followed the same approach we used in analyzing the patch residence time results (figure 3, table 1), fitting mixed-effects models with subject identity coded as a random effect. Including subject identity as a random effect accounts for the dependence that arises when taking multiple measurements from the same subject and thereby prevents the inflation of type-1 false-positive errors that can otherwise occur.

We used this approach to replace all comparisons that were previously based on t-tests and updated the text describing these comparisons accordingly. The overall pattern of results was unchanged, except for several of the comparisons of movement properties between male and female rats (figure 6) which are not significant when account for repeated measures. This strengthens our general conclusion (that differences in how male and female rats move through space cannot fully explain differences in their patterns of decision making), as it shows there is less evidence for sex differences in movement in the first place.

We have clarified our use of mixed-effects models to account for repeated measure in the methods section, and added the following reference discussing applications for mixed-effects models in neuroscience studies:

Yu, Z., Guindani, M., Grieco, S. F., Chen, L., Holmes, T. C., & Xu, X. (2022). Beyond t test and ANOVA: applications of mixed-effects models for more rigorous statistical analysis in neuroscience research. Neuron, 110(1), 21-35.

2. One of the authors' main goals with their new paradigm is to incorporate a spatial and movement component to foraging tasks that reflects natural foraging. While many aspects of their task fulfill this goal, I would argue that their "travel time" component does not. Rather than traveling a farther distance, rats sit in a small chamber for 0s or 60s of time. How is this different than operant chamber based foraging tasks that include such a delay?

We agree that simulating travel costs using a delay isn’t a perfect match to natural situations. We think our apparatus is nevertheless an improvement; rats do move between three distinct spatial contexts, each of which is larger than the typical operant box. Choosing to leave a patch entails actually leaving one part of the apparatus, travelling through the middle corridor, and arriving to a completely distinct location. This does not perfectly capture travelling between patches in a natural context, but it comes closer than, for instance, having rats press one lever to “stay” in a patch and different lever to “leave”, all the while remaining in the same physical location.

We have added to the discussion paragraph describing limitations of the task, noting that an interesting comparison for future studies would be to measure the impact of the delay (as used in the present work) with a version of the task that requires physical movement (using a running wheel or treadmill, for instance):

*Relatedly, simulating the cost of traveling between patches by imposing a delay is convenient, but clearly neglects the physical effort component that characterizes real-world travel. Developing a version of the task that requires actual movement to switch between patches (using a treadmill or exercise wheel, for instance) would make an interesting comparison with the present, delay-based task.*

3. Did the authors test for any order effects? For example, if on session A they had a long travel time and a slow depletion rate, I might expect them have a longer patch residence duration for session B. It would be informative to see if reward schedule/travel time for the previous session had any effect on patch residence duration.

We thank the reviewer for this suggestion, as we had not previously looked for session history effects. To answer this question we fit a mixed-effects model to examine how patch residence time was influenced by travel time, depletion rate, and initial reward rate, both for the current session parameters and those of the five preceding sessions. As with our previous analysis, this model included sex and side as fixed effects and subject identity as a random effect.

Standardized coefficient estimates are plotted in figure 4. Session 0 corresponds to the current session, and negative values for session number denote previous sessions. The current session travel time (β = 0.55, p = 2.02 × 10-54), patch depletion rate (βcurrent patch = -0.19, p = 7.92 × 10-10; βopposite patch = 0.09, p = 0.003), and initial reward rate (βcurrent patch = 0.15, p = 3.54 × 10-6; βopposite patch = -0.07, p = 0.02) all significantly influenced patch residence time. The travel time from the previous session also significantly influenced patch residence time (β = 0.11, p = 0.001). The effects of patch depletion rate and initial reward rate were limited to current session values only, with no significant influence of previous values for these variables. These results suggest that travel time had a lingering effect on behavior, indicating that rats integrated their estimates of some environmental statistics over a timescale longer than a single session.

4. For Figure 5E, I'm a bit confused about the heat plot. As it is, it appears that the most time a rat spent in one place over an 1800 s session (30 min) is 4 s in the corners. This seems surprising low, especially given that they spent almost no time in the large center portions of the chambers. Is this because rats (which are larger than the 1 cm squares used in the analysis) were only counted as 1 cm in the video tracking software (e.g. perhaps this is only tracking where their nose is)?

The reviewer is correct that the tracking system only marks the rat’s location with a single point corresponding to its center of mass. This fact, combined with the large number of bins that tracking data were divided into (to provide good resolution for where rats were spending time) means that the total occupancy within each bin is low.

Reviewer: 2

Comments to the Author

The present work aims to establish a patch-foraging model in rats. My comments to improve the manuscript are minor.

1) The strain of rat used should be described in the Abstract.

Agreed, done.

2) How the data was analyzed needs to be further clarified/elaborated. Particularly, because the authors state that they used 10 animals across experiments; yet the data points within the bar-figures exceeds the number of animals used.

We have added additional clarification to the methods, noting that subjects were tested multiple times across a range of different session parameters, and linear mixed effects models were used to analyze the data while appropriately accounting for repeated measurements taken from the same rat:

*All models throughout the paper were linear mixed effects models that were fit using the Matlab function fitlme. These models allow us to quantify how a dependent variable of interest is modulated by a set of predictors. Importantly, mixed-effects models allow for subject identify to be included as a random factor, which accounts for the dependence in data arising from repeated measurements within individual rats (Yu et al., 2022). Thus, for all models reported throughout the paper, subject identity was included as a random effect.*

3) The authors should briefly discuss how food-type may influence their sex-specific results, because females display higher preference for sweetened-fat when compared to males [see Maric et al., Frontiers in Nutrition, 9, 828522, 2022; and Castro et al, Dev Psychobiology, 64(8):e22345, 2022]. In other words, would the results be similar or different if the pellets had different caloric and/or sweet-fat content? Is it possible that the animal’s motivation to forage may be influenced by such factors?

We thank the reviewer for pointing us to this literature. While it is difficult to say definitely how a different reinforcer would have affected our results, we agree that this is an important issue to consider for future work. We have added the following text to the discussion paragraph that summarizes limitations of the current work:

*In addition, testing a wider range of outcomes would help establish the generality of these results; for instance, there is evidence for sex differences in motivation to earn outcomes with certain combinations of nutrients (Castro et al., 2022; Maric et al., 2022). Such differences could influence foraging strategies measure on the task.*

4) Given that the authors are establishing a new behavioral model, it may be useful to briefly address the type of validity their model captures best in the discussion section of the paper.

We feel this task builds on ethological validity compared to other tests of decision making. We have added the following section to the discussion to build on this point:

*Testing decision making in a way that mimics the natural structure of foraging decisions increases the ethological validity of the paradigm by tailoring the relevant sensorimotor features of the task to match what animals would typically experience in natural settings (Juavinett et al., 2018). For all of these reasons, well-validated laboratory tests of foraging behavior are a valuable tool for understanding the neurobiology of decision making.*
